# Lactylation signature identifies liver fibrosis phenotypes and traces fibrotic progression to hepatocellular carcinoma

**DOI:** 10.3389/fimmu.2024.1433393

**Published:** 2024-08-27

**Authors:** Lin-na Li, Wen-wen Li, Lu-shan Xiao, Wei-nan Lai

**Affiliations:** ^1^ Department of Endocrinology and Metabolism, Nanfang Hospital, Southern Medical University, Guangzhou, Guangdong, China; ^2^ Guangzhou Wondfo Health Science and Technology Co., Ltd, Guangzhou, China; ^3^ Department of Infectious Diseases, Nanfang Hospital, Southern Medical University, Guangzhou, China; ^4^ Department of Rheumatology and Immunology, Nanfang Hospital, Southern Medical University, Guangzhou, China

**Keywords:** liver fibrosis, lactylation, machine learning, immune infiltration, hepatocellular carcinoma

## Abstract

**Introduction:**

Precise staging and classification of liver fibrosis are crucial for the hierarchy management of patients. The roles of lactylation are newly found in the progression of liver fibrosis. This study is committed to investigating the signature genes with histone lactylation and their connection with immune infiltration among liver fibrosis with different phenotypes.

**Methods:**

Firstly, a total of 629 upregulated and 261 downregulated genes were screened out of 3 datasets of patients with liver fibrosis from the GEO database and functional analysis confirmed that these differentially expressed genes (DEGs) participated profoundly in fibrosis-related processes. After intersecting with previously reported lactylation-related genes, 12 DEGs related to histone lactylation were found and narrowed down to 6 core genes using R algorithms, namely S100A6, HMGN4, IFI16, LDHB, S100A4, and VIM. The core DEGs were incorporated into the Least absolute shrinkage and selection operator (LASSO) model to test their power to distinguish the fibrotic stage.

**Results:**

Advanced fibrosis presented a pattern of immune infiltration different from mild fibrosis, and the core DEGs were significantly correlated with immunocytes. Gene set and enrichment analysis (GSEA) results revealed that core DEGs were closely linked to immune response and chemokine signaling. Samples were classified into 3 clusters using the LASSO model, followed by gene set variation analysis (GSVA), which indicated that liver fibrosis can be divided into status featuring lipid metabolism reprogramming, immunity immersing, and intermediate of both. The regulatory networks of the core genes shared several transcription factors, and certain core DEGs also presented dysregulation in other liver fibrosis and idiopathic pulmonary fibrosis (IPF) cohorts, indicating that lactylation may exert comparable functions in various fibrotic pathology. Lastly, core DEGs also exhibited upregulation in HCC.

**Discussion:**

Lactylation extensively participates in the pathological progression and immune infiltration of fibrosis. Lactylation and related immune infiltration could be a worthy focus for the investigation of HCC developed from liver fibrosis.

## 1 Introduction

Liver fibrosis, or hepatic fibrosis, often begins with a fibrous scar arising from extracellular matrix (ECM) accumulation, especially crosslinked collagens type I and type III (1). Fibrous scar formation, in replacement of damaged normal tissue, is constantly triggered following chronic liver injury. Viral infection (2), alcohol abuse (3), metabolic dysfunction-associated steatohepatitis (MASH), and metabolic-associated fatty liver disease (MAFLD) foster persistent activation of inflammatory response and fibrogenesis, leading to the development of liver fibrosis (4). Fibrosis progression from reversible to advanced stage may deteriorate into cirrhosis, liver failure, and portal hypertension (5). Cirrhosis is one of the intermediate processes of liver diseases developing hepatocellular carcinoma (HCC), no matter whether it originated from alcohol abuse, hepatitis virus infection, or metabolic dysfunction (6). The last decades have witnessed an epidemic of liver fibrosis, despite social efforts on HBV/HCV (hepatitis B virus and hepatitis C virus) delimitation. Non-viral etiology for liver fibrosis increased due to superfluous living supplies. Infection rates of hepatitis virus, prevalence of metabolism-related liver diseases, and per capita consumption of alcohol in China surpassed those in other countries and regions; moreover, liver fibrosis and cirrhosis largely account for hospitalization of patients with liver disease (7). Fibrotic progression to liver failure can only be treated by liver transplantation (8), highlighting the hierarchical management of patients with liver fibrosis. Therefore, reversing early-stage fibrosis and curbing advanced fibrosis from progressing into liver failure is one of the most urgent challenges for public health.

Recent studies underscore the roles of histone lactylation in various diseases (9–11). Lactylation, a novel histone acetylation that was newly defined in 2019, has emerged to the sight of researchers with its modulatory roles in inflammation, fibrosis, cell differentiation, and cancerous development (10). Lactylation can exert reparative (12) and injurious (13) functions concerning all kinds of immunocytes, stroma cells, and histiocytes (14–16). Immunocytes, in response to anti/pro-inflammatory and angiogenetic signals, undergo metabolic reprogramming and a drastic increase in glycolysis produces abundant lactide that fuels histone lactylation and subsequent modulation of gene expression (17).

Lactylation in liver fibrosis is under intensive study but far from fully elucidated. Liver fibrosis involves joint action from hepatic stellate cells (HSCs), immunocytes, and hepatocytes (18). HSC activation features dynamic glycolysis and entails histone lactylation for transcriptional activation of key genes sustaining fibrotic pathology (19, 20). However, lactylation of hepatocytes and its impact on the infiltration and activation of immune cells has been scarcely studied.

Enlightened by the prerequisite role of lactylation in HSC activation and fibrotic phenotypes, the study aims to comprehensively assess the activity and staging value of lactylation in liver fibrosis. Combining the Gene Expression Omnibus (GEO) database and documented lactylation-related genes (21–23), we screened differentially expressed genes (DEGs) with lactylation and tested their power to distinguish early and advanced fibrosis. Functional and enrichment analyses as well as immune infiltration analysis validated that the DEGs were closely related to fibrosis progression and immune infiltration.

## 2 Method and materials

### 2.1 Data sources

Raw gene expression and stage data of patients with liver fibrosis from three datasets, namely GSE130970, GSE84044, and GSE49541 were downloaded from the GEO database (http://www.ncbi.nlm.nih.gov/geo/). In GSE130970, fibrosis was staged by scoring tissue sections stained with Masson’s trichrome stain using the NIDDK NASH CRN staging system, and stages 1a, 1b, and 1c were classified as stage 1 (24). The dataset contained 23 control subjects, 28 fibrosis cases at stage 1, 9 at stage 2, 14 at stage 3, and 2 at stage 4, and gene expressions were detected by Illumina HiSeq 2500 (Homo sapiens) on the GPL16791 platform. GSE84044 provides data on 43 non-fibrotic livers, 20 fibrosis cases at stage 1, 33 at stage 2, 18 at stage 3, and 10 at stage 4 (25). The dataset applied the Affymetrix Human Genome U133 Plus 2.0 Array [HG-U133_Plus_2] on GPL570 for gene sequencing. A total of 40 control and fibrotic livers at stage 1 were grouped against 32 cases of fibrosis at stages 2 to 4 in GSE49541(GPL570[HG-U133_Plus_2] Affymetrix Human Genome U133 Plus 2.0 Array). Therefore, we defined stages F0 to F1 as mild fibrosis and stages F2 to F4 as advanced fibrosis. Datasets were merged and batch effects were adjusted using the limma and sva packages in R language. R version 4.2.2 was used for all analyses in this article. The FactoMineR and factoextra packages were utilized for principal component analysis (PCA) and plotting to visualize the adjustment on three-dimensional scatter plots.

To verify the generalization of core DEGs in fibrosis, GSE14323, GSE103580, GSE110147, and GSE150910 from the GEO database were collected. GSE14323 and GSE103580 provided liver specimen expression data of patients with HCV-related and alcohol-related cirrhosis, respectively. Gene expression of lung samples from patients with idiopathic pulmonary fibrosis and normal controls were gathered from GSE110147 and GSE150910.

Expression and survival data of patients with HCC (TCGA-LIHC cohort) were obtained from the TCGA website (https://www.cancer.gov/ccg/research/genome-sequencing/tcga).

### 2.2 Screening of differentially expressed genes

A linear model for microarray data (LIMMA) package (26) from R language was used for DEG screening. To improve the reliability of differentially expressed genes, probe sets for which the adjusted P was <0.05, and |logFC| was > 0.25 between mild and advanced fibrosis were defined as significantly differentially expressing.

### 2.3 Functional analyses of DEGs and gene set enrichment analysis

In order to investigate the biological functions of DEGs, ClusterProfiler package (27) was utilized for functional analyses. The analyses incorporated Gene Ontology (GO) and Kyoto Encyclopedia of Genes and Genomes (KEGG), and the GO category delineated functional pathways into three aspects including biological processes (BP), molecular functions (MF), and cellular components (CC). The P-value was adjusted using the Benjamini–Hochberg approach or FDR for multiple testing corrections. The threshold was set at FDR<0.05.

In gene set enrichment analysis (GSEA) for core DEGs, the clusterProfiler package was utilized to calculate the enrichment score of pathways with the given genes.

### 2.4 Selection of core DEGs

After intersection with the lactylation-related gene list, 12 candidates were subjected to two machine learning algorithms, random forest (RSF) and support vector machine (SVM), for characteristic gene selection. The harvested six genes were further tested by least absolute shrinkage and selection operator (LASSO), another machine learning algorithm characteristic of dimension reduction. LASSO analysis was implemented with a turning/penalty parameter utilizing a 10-fold cross-verification via the glmnet package (28). Receiver operating characteristic (ROC) curves and the area under the curve (AUC) were used to estimate the diagnostic efficacy.

In detail, to model the degree of liver fibrosis, we used the “glmnet” function in LASSO regression and the variable type was binomial. When performing 10-fold cross-validation using the cv.glmnet function, the “coef” function was utilized to extract the coefficients of the model. The parameter s=cvfit$lambda.min specifies the coefficient corresponding to the minimum lambda value selected using cross-validation.

This article uses two machine learning methods to screen key genes, SVM and RSF. SVM and RSF are commonly used machine learning methods. SVM is a supervised learning algorithm primarily used for classification and regression problems. Its basic principle is to find the optimal boundary between data points (hyperplane), which can maximize the boundary distance between different categories. SVM uses kernel functions to map data to high-dimensional space to handle nonlinear separable problems. In gene screening, SVM can be used to distinguish samples with different biological characteristics or phenotypes by identifying which genes are most important for classification. RSF is an ensemble learning method based on decision tree construction. It improves the accuracy and robustness of the model by constructing multiple decision trees (forests) and voting or averaging their results. Each decision tree uses a random subset of the dataset during training (achieved through self-sampling), which increases model diversity and reduces the risk of overfitting. An important feature of RSF is its feature importance assessment, which can identify the genes that impact classification most greatly. In gene screening, RSF can be used to evaluate the contribution of genes to sample classification and select key genes through feature importance scores. Combining SVM and RSF can improve the accuracy of predictions.

### 2.5 Immune cell infiltration analysis

Single-sample gene set enrichment analysis (ssGSEA) was implemented to analyze the immune infiltration based on the expression profiling of 29 immunity-relevant signatures. The analyses were composed of reciprocal relevance between different types of immune cells, differences in immune infiltration between mild and advanced fibrosis, and correlation of immune cells with core DEGs.

The ssGSEA function in the Gene Set Variation Analysis (GSVA) package evaluates the degree of association between a single sample and a predefined gene set. It is comprised of the following key steps:

#### 2.5.1 Immune cell infiltration analysis

Pre-processing of gene expression data: First, gene expression data must be converted into a format suitable for GSVA analysis.

#### 2.5.2 Preparation of gene sets

The gene sets used in this article represent cell types and pathways.

#### 2.5.3 Non-parametric pathway enrichment analysis

GSVA uses nonparametric methods to evaluate the degree of association between each sample in the gene expression profile and every certain gene set, and converts the associations into a continuous score.

#### 2.5.4 Kernel density estimation

GSVA estimates the expression distribution of gene sets through kernel functions (Gaussian kernel in this paper).

#### 2.5.5 Empirical cumulative distribution function

GSVA uses eCDF to evaluate the position of gene sets in gene expression ranking lists.

#### 2.5.6 Enrichment score

ES was calculated by taking the maximum absolute value of the difference between the cumulative distribution function of all genes in the gene set and the cumulative distribution function of the entire gene set.

#### 2.5.7 Statistical test

Finally, linear models and empirical Bayesian methods are used to perform statistical tests on the ES to determine whether the enrichment of the gene set is statistically significant.

The p-value ranged from 0 to 1, and less than 0.05 was considered significant.

### 2.6 Unsupervised hierarchical clustering

The normalized expression microarray data for each patient were collected and subjected to unsupervised hierarchical clustering with the ConsensusClusterPlus package in R.

### 2.7 Gene set variation analysis

KEGG and Reactome pathways were downloaded from the MSigDB database as the reference set. The GSVA scores of each pathway were calculated using the ssGSEA function in the GSVA package from R. The GSVA score denoted the degree of absolute enrichment of each gene set, and was compared across two clusters using the limma package.

### 2.8 Construction of regulatory network

Regulator data concerning miRNA and transcriptional factors were obtained from the regnetwork database(https://regnetworkweb.org/) for upstream prediction of core DEGs. The regulatory network was constructed using Cytoscape software.

### 2.9 Statistical analysis

All the statistical analyses were performed using R-4.1.3. Heatmaps were plotted using R package “pheatmap”. The KM method was performed using the R package “survminer”. LASSO analysis was performed using the R package “glmnet”. The KM plots, violin plots, volcano plots were plotted using the R package “ggplot2”. For comparison between two groups, Student’s t-test was performed.

## 3 Results

### 3.1 Integrating microarray datasets of liver fibrosis

**Figure 1:**
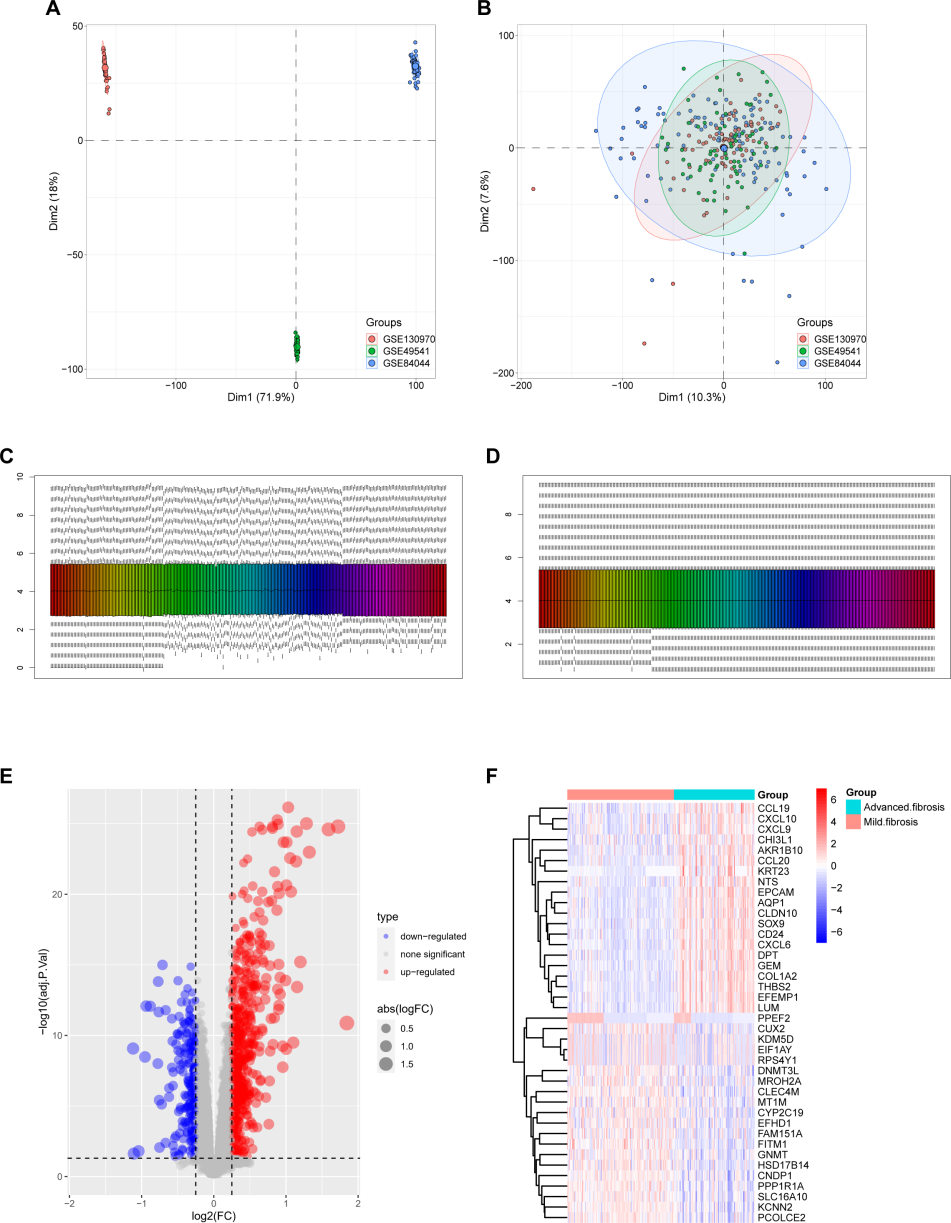
Merging the database and screening differential expressed genes (DEGs). **(A)** Principal Component Analysis (PCA) of samples from the three databases before data merging. **(B)** PCA of 274 samples covering 16,243 genes after data merging using the FactoMineR and factoextra packages from R. **(C, D)** sample distribution before **(C)** and after **(D)** homogenization of the datasets using the preprocessCore package of R language. **(E, F)** DEGs were screened using the limma package under the standard of adj.P.Val<0.05, and |logFC| > 0.25. Upregulated genes were plotted in red and downregulated genes in blue in a volcano plot **(E)**. The heatmap **(F)** displays a group of genes differentially expressed in mild and advanced fibrosis.

The three liver fibrosis datasets (GSE130970, GSE84044, and GSE49541) were incorporated into the study and merged using the limma and sva algorithms to remove batch effects. Distribution patterns of the fibrotic cases before and after normalization were visualized using principal component analysis (PCA) (**Figures 1A, B**) and box plots (**Figures 1C, D**).

### 3.2 Differentially expressed genes between mild and advanced liver fibrosis

After homogenization, the samples were subjected to variance analysis using the limma package. Distinct gene expression patterns between mild and advanced fibrosis were identified (adj.P.val < 0.05, and |log2FC| > 0.25), with 629 DEGs upregulated and 261 downregulated for liver fibrosis, as shown in the volcano plot and heatmap (**Figures 1E, F**).

### 3.3 Enrichment analyses of DEGs

**Figure 2:**
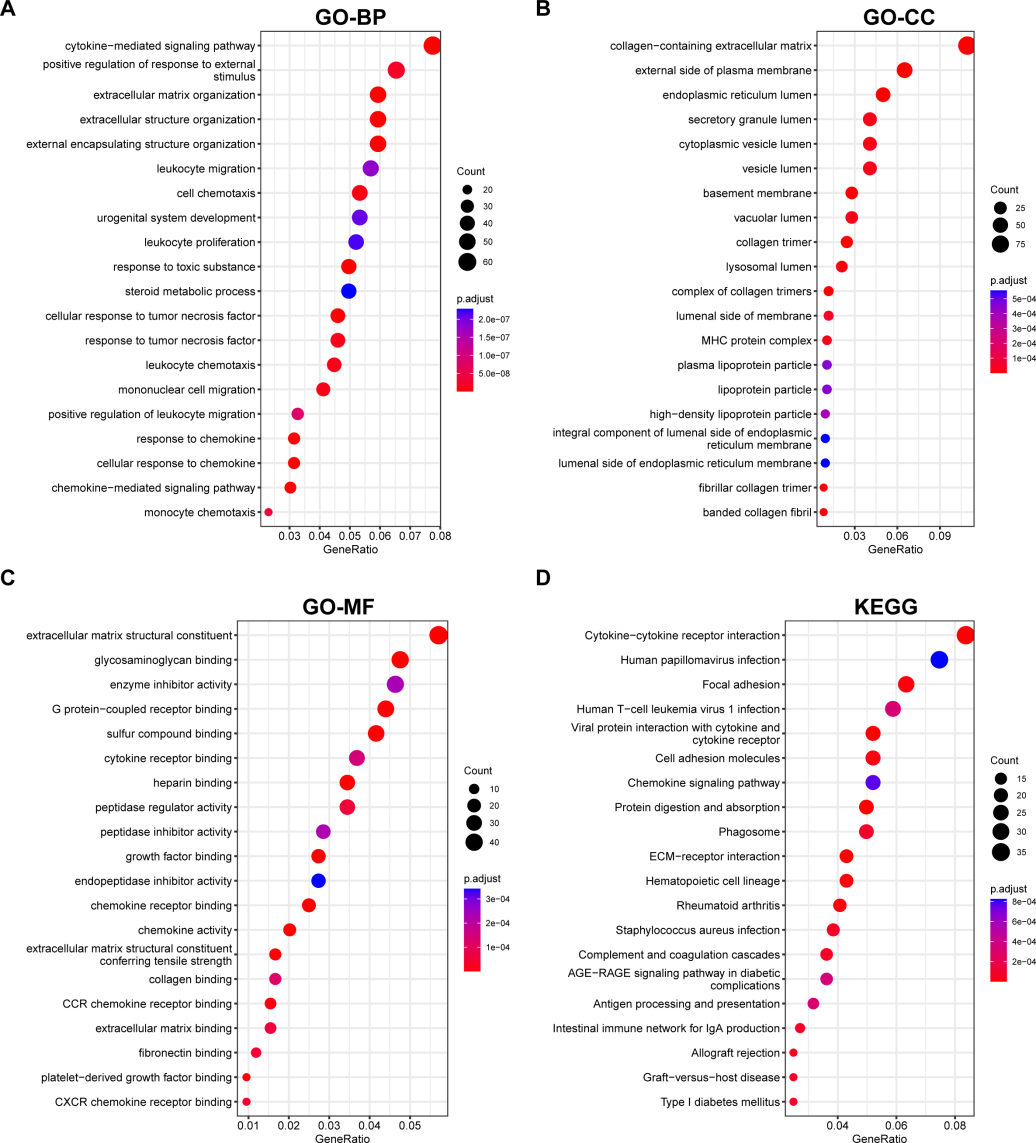
GO Annotation and KEGG Enrichment analyses of DEGs. **(A–C)** GO annotation of the DEGs in association with annotated **(A)** biological process (BP), **(B)** cellular component (CC), and **(C)** molecular function (MF). **(D)** demonstration of KEGG enrichment results. Pathways were ranked according to their GeneRatio, and the sizes of the bubbles represent the number of enriched genes, and the colors represent *p* values.

Next, we performed pathway enrichment analyses on the DEGs of liver fibrosis. GO analysis revealed that these DEGs were enriched in fibrotic processes, such as “cytokine–mediated signaling”, “cell chemotaxis”, and “extracellular matrix organization” (**Figures 2A–C**). KEGG analysis highlighted their involvement in “cytokine–cytokine receptor interaction”, “chemokine signaling pathway”, “ECM–receptor interaction”, etc (**Figure 2D**). Enrichment for these pathways suggested that these DEGs were associated with chemokine signaling and excessive production of extracellular matrix, which are responsible for liver fibrosis.

### 3.4 Lactylation underscores the core predictive DEGs in fibrotic livers

**Figure 3:**
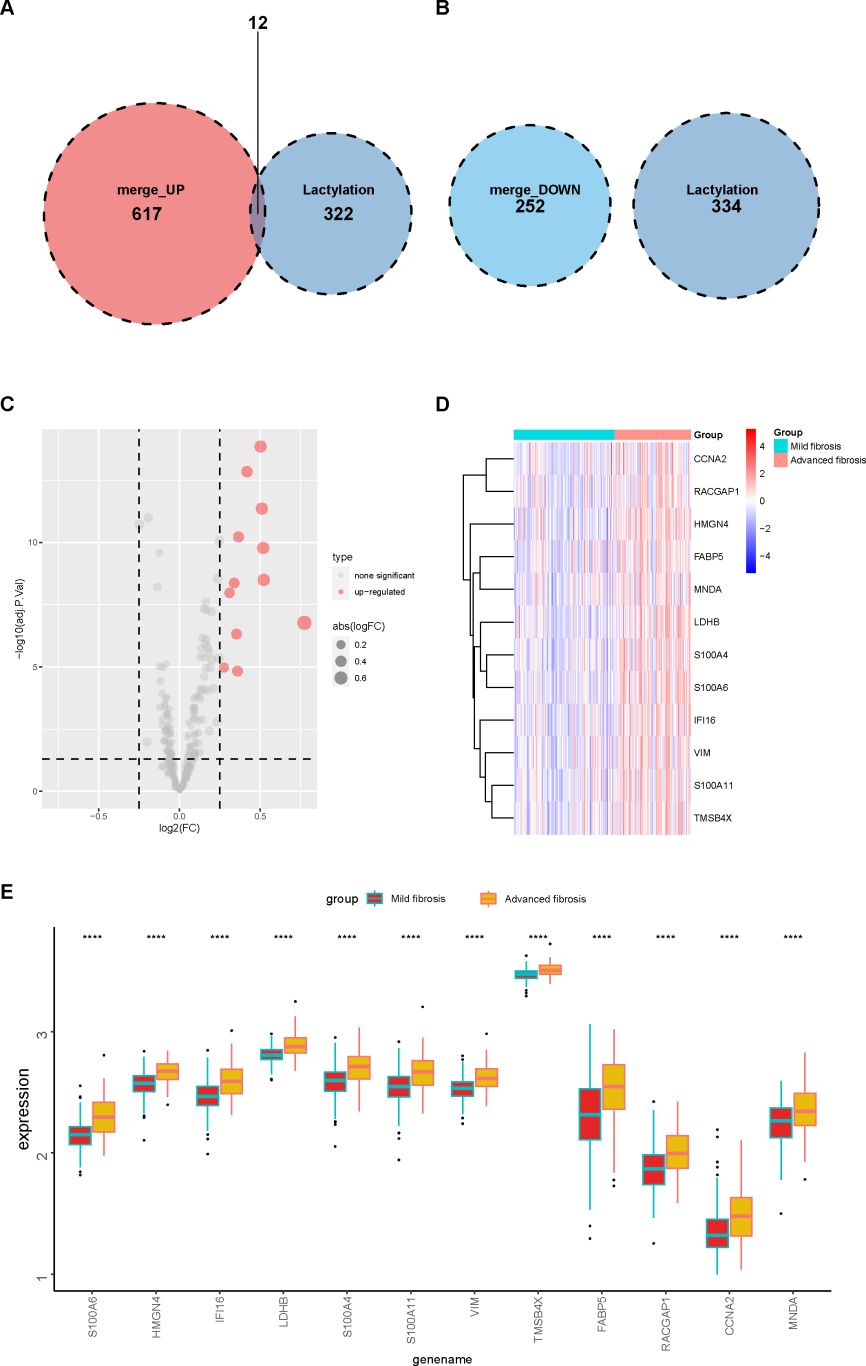
Lactylation of DEGs in mild and advanced fibrotic livers. **(A, B)** Venn plot displaying the intersection of upregulated **(A)** and downregulated **(B)** DEGs with lactylation–related genes. **(C)** a volcano plot displaying the expressions of lactylation–related genes in mild and advanced fibrotic livers. **(D, E)** heatmap **(D)** and boxplot **(E)** displaying the expression patterns of the 12 lactylation–related genes in mild and advanced fibrotic livers. ****p<0.001.

By intersecting these DEGs with 336 lactylation–modified genes (21–23), we found that among the upregulated DEGs, 12 genes were modulated by lactylation (**Figure 3A**), while none of the downregulated DEGs were related to lactylation (**Figure 3B**). We further analyzed the 336 lactylation–related genes in patients with or without liver fibrosis and the results validated that the expression level of only these 12 upregulated DEGs fluctuated between fibrotic and normal livers (**Figure 3C**). The 12 genes included S100A6, HMGN4, IFI16, LDHB, S100A4, S100A11, VIM, TMSB4X, FABP5, RACGAP1, CCNA2, and MNDA, and their expression patterns in advanced fibrosis were distinct from mildly fibrotic livers (**Figures 3D, E**). Thus, these genes might have crucial functions in the pathology of liver fibrosis.

**Figure 4:**
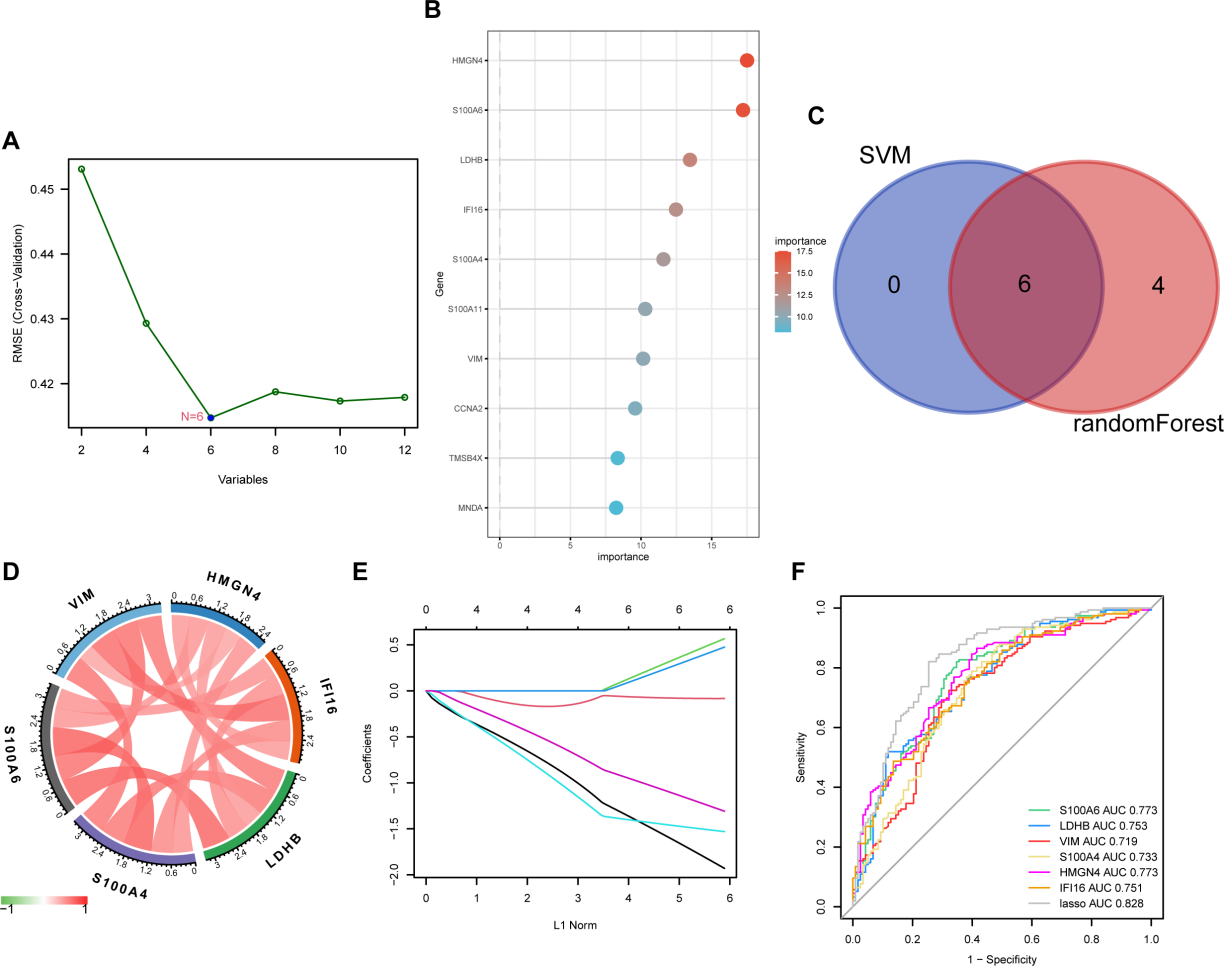
Key DEG screening using machine learning. **(A)** key DEGs screened by SVM (support vector machine) using the kernlab package from R. **(B)** key DEGs elected by random forest using the randomForest package of R and ranked in order of their importance. **(C)** key DEGs selected by SVM and random forest were intersected and six candidates were obtained. **(D)** chord diagram displaying the correlation of six core DEGs. Positive correlations were plotted in red and negative in green. **(E)** the LASSO coefficient profiles of the six core DEGs in predicting liver fibrosis plotted by the glmnet package from R. **(F)** ROC (receiver operating characteristic) curve of the six core DEGs and their LASSO model in predicting disease occurrence plotted using the pROC package from R. AUC, area under the curve.

We applied two machine algorithms to screen out signature genes from the 12 lactylation–related DEGs in liver fibrosis. Six candidates of predictive value for liver fibrosis were selected by SVM (**Figure 4A**) and ten by random forest (**Figure 4B**). The harvested key DEGs were further intersected (**Figure 4C**), which returned six core candidates, namely S100A6, HMGN4, IFI16, LDHB, S100A4, and VIM, with significantly and reciprocally positive correlations (**Figure 4D**). We constructed a LASSO regression model based on the six core genes (**Figure 4E**), which manifested better predictive power than each single DEG as shown by the ROC curve (**Figure 4F**), with the AUC reaching 0.828 in LASSO model and ranging from 0.719 (VIM) to 0.773 (S100A6 and HMGN4).

### 3.5 Liver fibrosis progression involves variations in immune infiltration and gene expression

**Figure 5:**
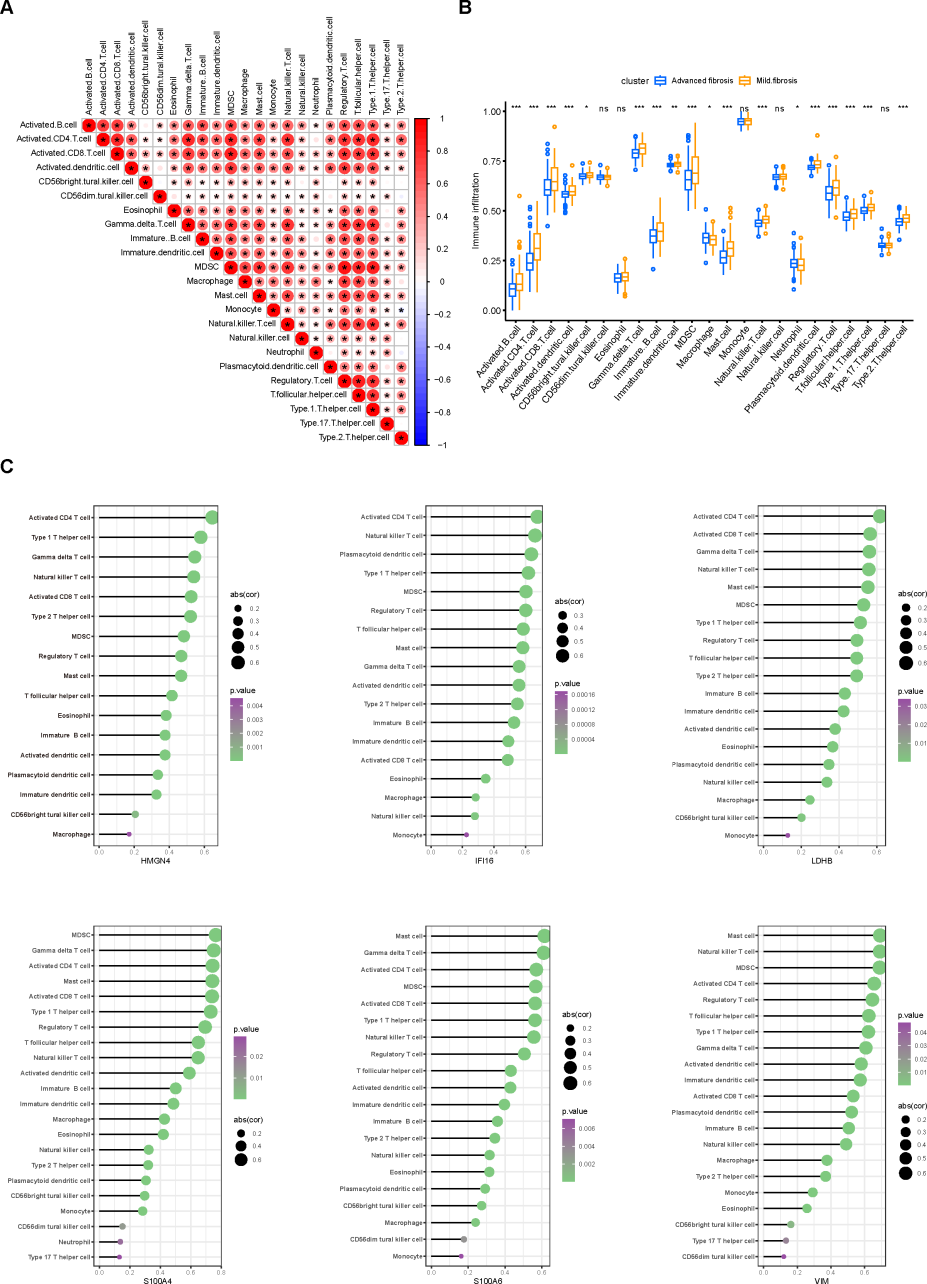
Immune infiltration in fibrotic livers and its correlation with core DEGs. **(A)** correlation heatmap displaying the correlation of various immune cells in the aspect of their proportion in fibrotic livers. **(B)** differences in immunocyte infiltration between mild and advanced fibrosis. Ns, not significant, ^*^ *p*<0.05, ^**^ *p*<0.01, ^***^ *p*<0.001. **(C)** associations between the degree of immune infiltration and each core DEG were plotted using the ggplot2 package on R. Immunocytes with *p* values less than 0.05 are displayed, sizes of the bubbles represent correlation coefficients and colors represent *p* values.

We continued to investigate the immune infiltration in the fibrotic livers. Cross infiltration of all kinds of immune cells revealed the intertwined inflammatory status along fibrotic progression (**Figure 5A**), especially the strongly significant coexistence of activated B cells with activated CD4, CD8 T cells, and γδ T cells that are responsible for the pro-inflammatory process, as well as the myeloid-derived suppressor cells (MDSCs) and regulatory T cells that exert immunoregulatory functions. Moreover, infiltration of immunocytes presented distinctive alterations along the progression of liver fibrosis (**Figure 5B**). Most immunocytes, including activated B cells, activated CD4 and CD8 T cells, dendritic cells, γδ T cells, MDSCs, and regulatory T cells increased while macrophages and neutrophils decreased along with fibrosis advancement. Moreover, infiltration of different immunocytes was associated with the core DEGs (**Figure 5C**). Typically, activated CD4 T cells were the top immunocyte correlated with HMGN4, IFI16, and LDHB, while MDSCs and mast cells were closely related to S100A4, S100A6, and VIM. In addition, immunocytes that distinctively infiltrated in mild and advanced fibrosis also exhibited significant correlations with the core DEGs. These results suggested that not only did immune infiltration participate in fibrotic progression, but also certain core genes might be involved in the proportion variation of immunocytes.

### 3.6 Phenotyping power of the core DEGs in liver fibrosis

**Figure 6:**
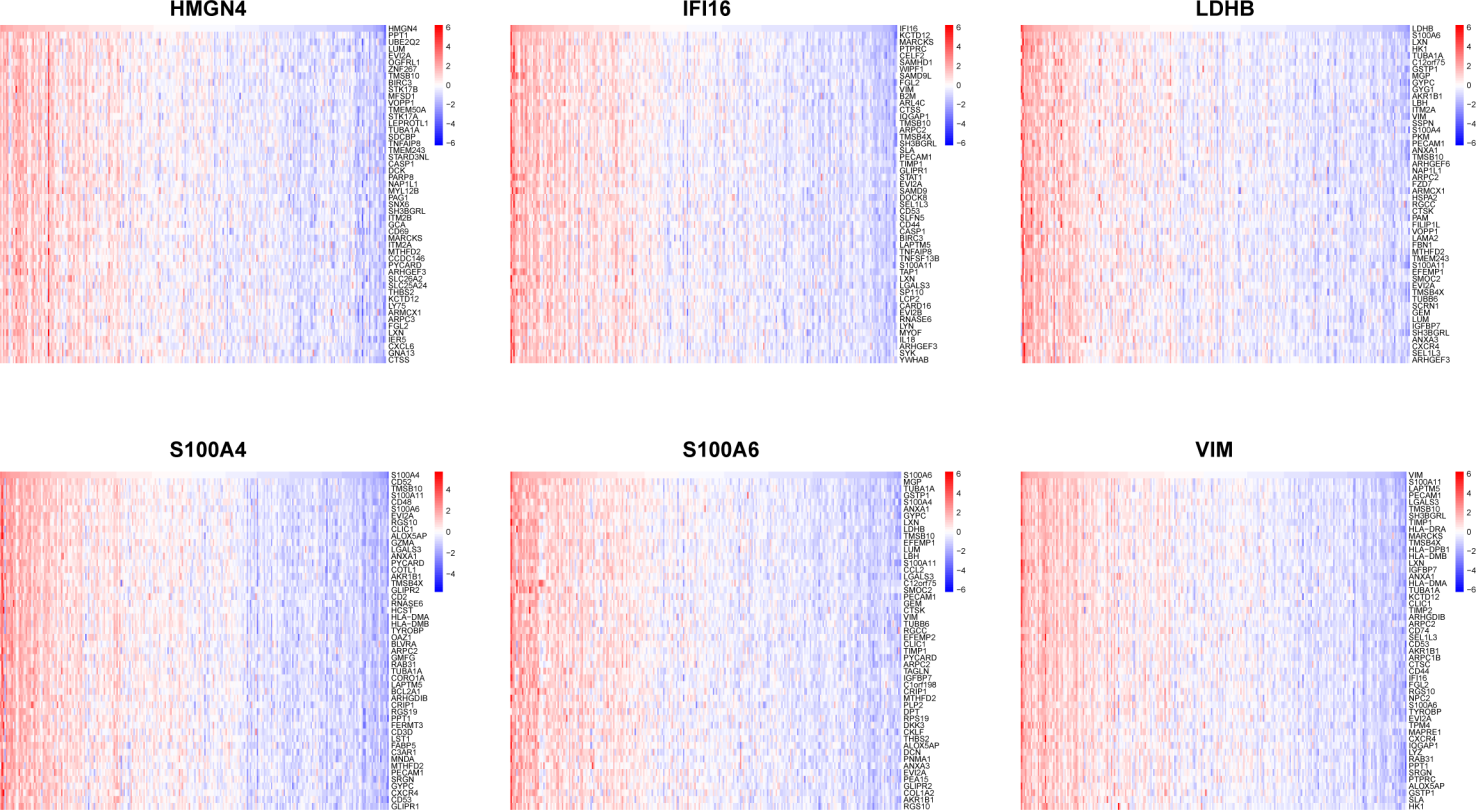
Association of core DEGs with genome variants. Correlation heatmaps illustrating the association between a single core DEG and 50 top-related genes.

**Figure 7:**
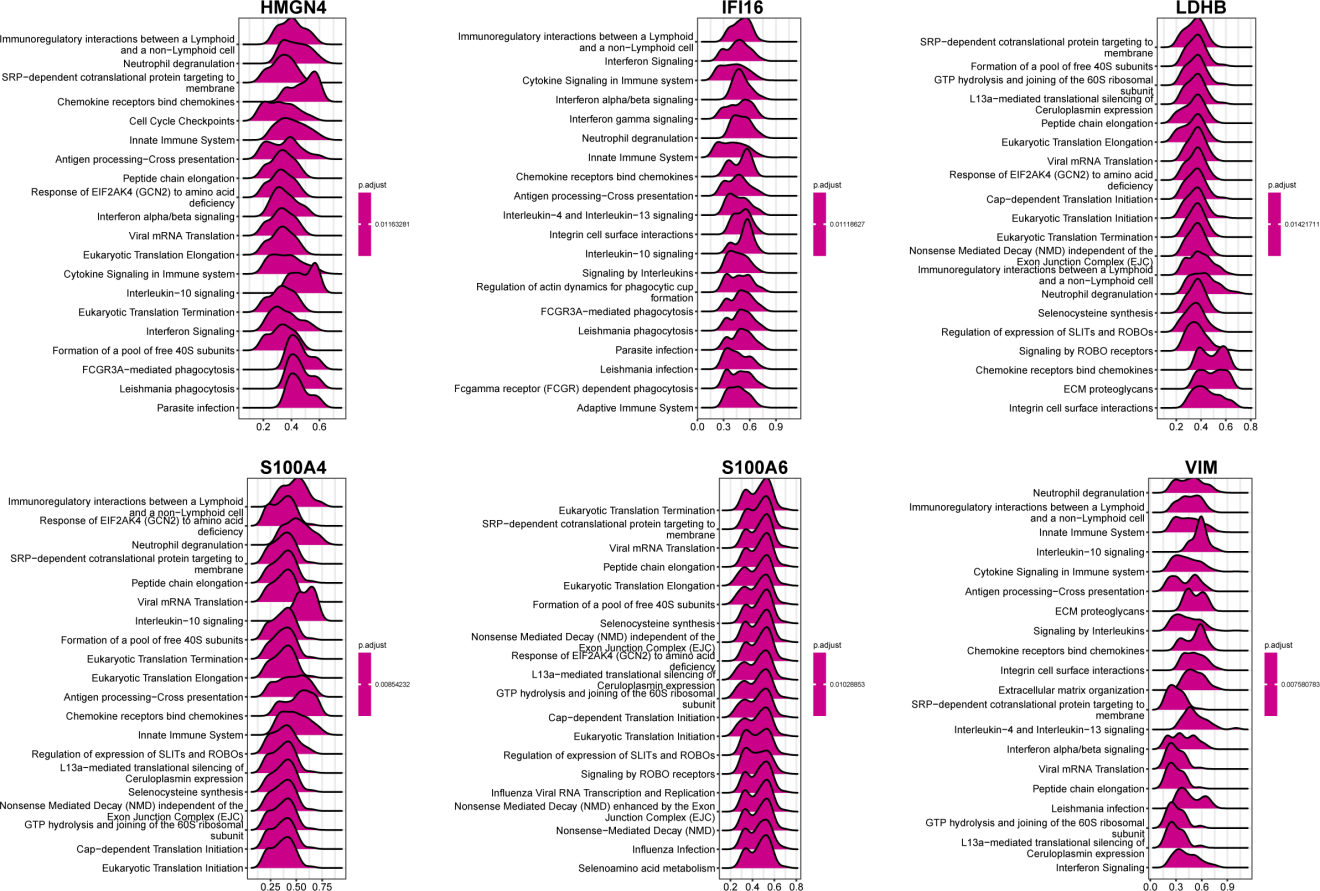
GSEA of genes correlated with core DEGs. GSEA was performed based on the genes selected by correlation analysis using clusterProfiler from R. Top20 Reactome pathways of GSEA results are plotted with the enrichment score on the x-axis.

In order to catalog liver fibrosis with core DEGs, we screened genes notably related to each core DEG (**Figure 6**), which were sequentially subjected to GSEA (**Figure 7**). Per the aforementioned results that these DEGs were linked to immune infiltration in liver fibrosis (**Figure 5**), genes screened by their relationship with core DEGs were enriched in pathways involving immune responses and chemokine signaling including “immunoregulatory interactions between a Lymphoid and a non-Lymphoid cell”, “neutrophil degranulation”, “interferon signaling”, “neutrophil degranulation”, “cytokine signaling in immune system”, and “chemokine receptors bind chemokines”.

**Figure 8:**
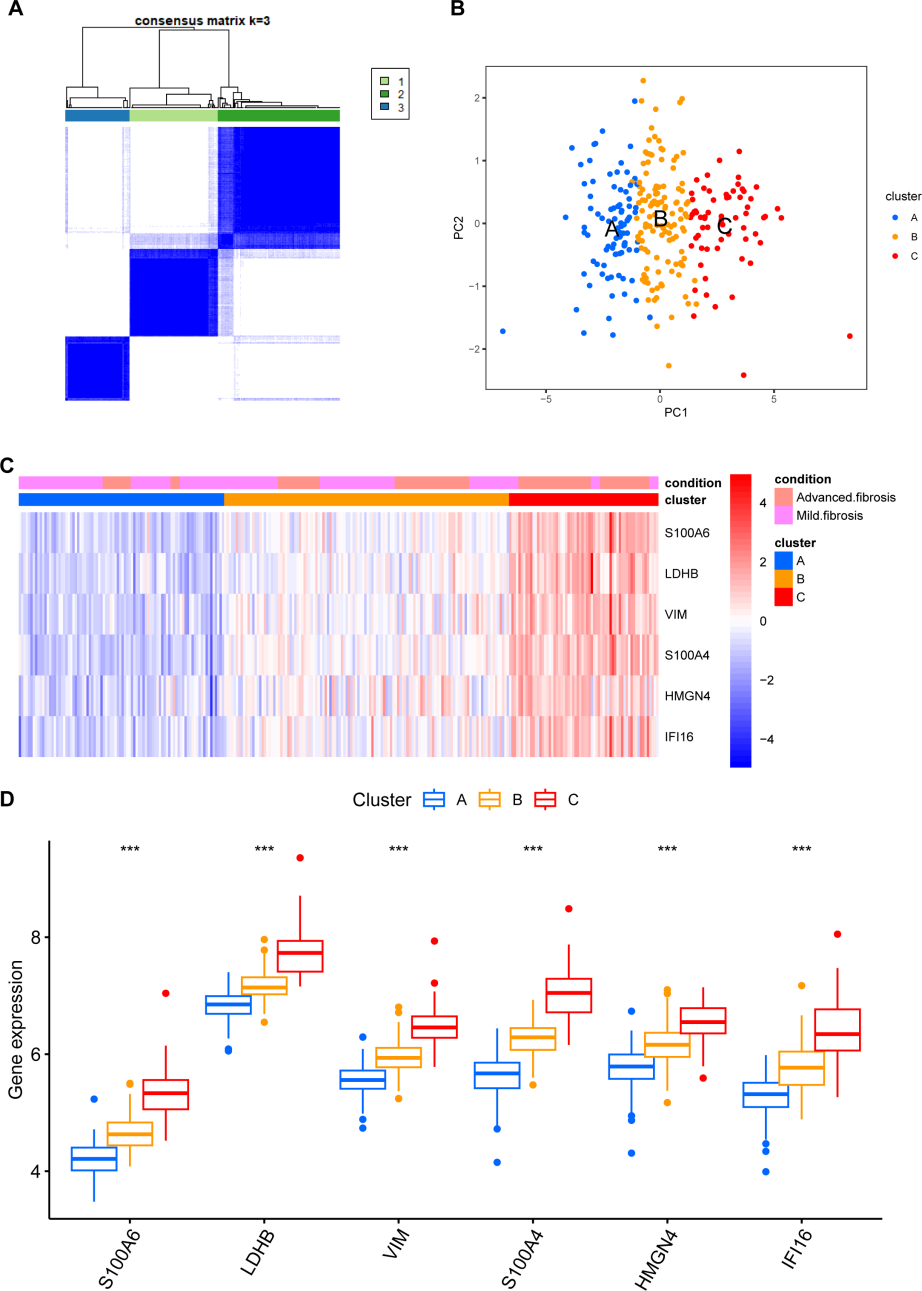
Phenotype clustering by the expression of core DEGs. **(A)** consensus clustering on liver fibrosis samples based on the six core DEGs using the ConsensusClusterPlus package from R. **(B)** PCA of the sample distribution across different phenotypes. **(C)** heatmap showing the association between gene expression and different phenotypes plotted by pheatmap from R. **(D)** expression distinction of core DEGs across different phenotypes. ^***^ *p*<0.001.

**Figure 9:**
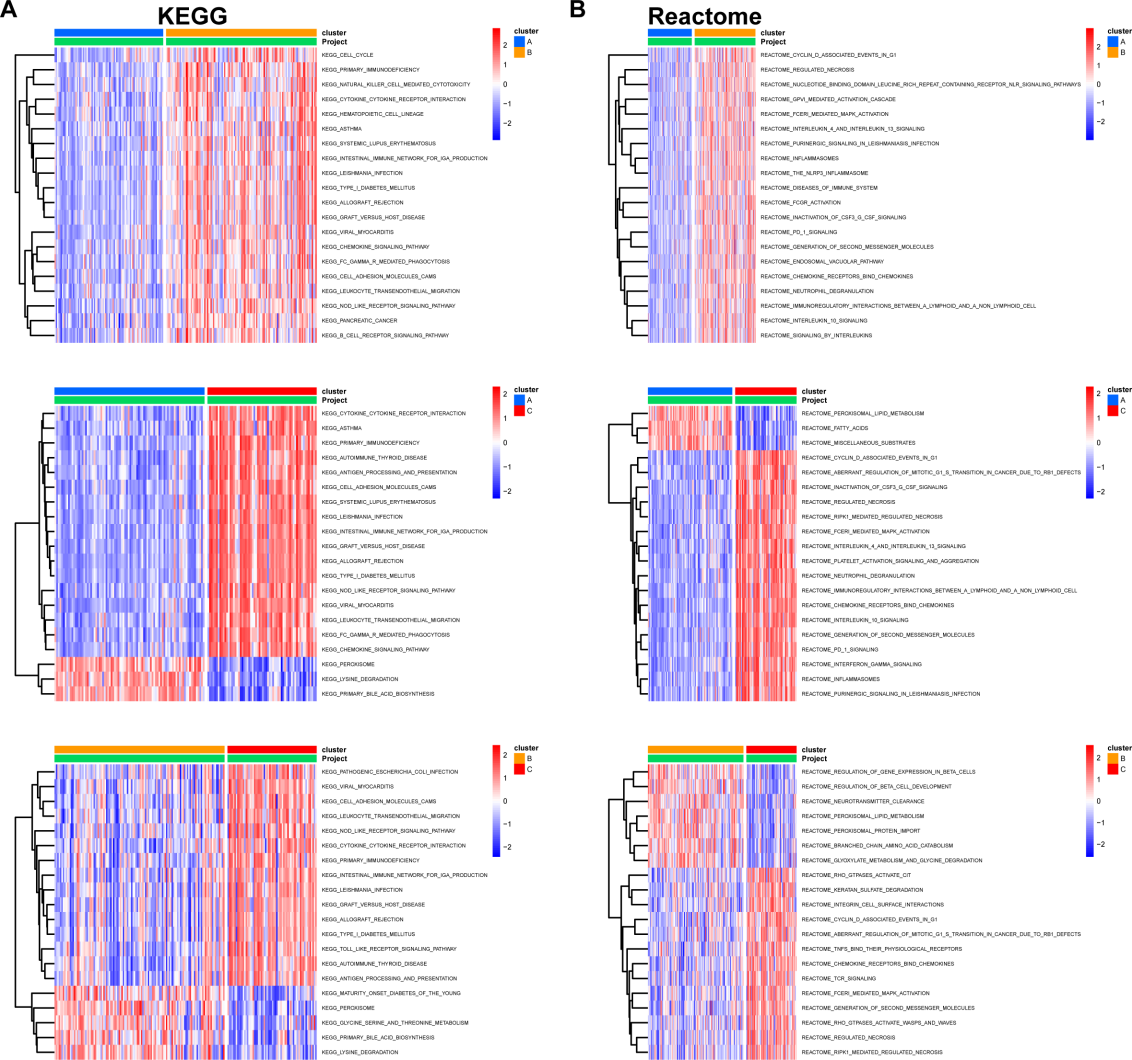
Pairwise GSVA between different clusters. KEGG **(A)** and Reactome **(B)** pathways enriched for indicated clusters. GSVA package in R was used to compare pathway enrichment reciprocally between each two clusters. Pathways with significant differences were plotted on heatmaps using the pheatmap package from R. The color columns represent enrichment scores for the pathways in each cluster.

We then classified 274 samples from three databases into three phenotypes according to their expression of core DEGs (**Figure 8A**). The PCA results confirmed that these samples were discriminately stratified (**Figure 8B**). In detail, advanced fibrosis accumulated in clusters B and C more than in cluster A; furthermore, cluster A featured low expression of the core DEGs, which were moderately upregulated in cluster B and drastically increased in cluster C (**Figures 8C, D**). GSVA revealed that pathways related to immune response, such as “primary immunodeficiency”, and “cytokine receptor interaction” were enriched in clusters B and C (**Figure 9A**); also, clusters A and B were characterized by activated lipid metabolism and signaling, including pathways involving “peroxisomal lipid metabolism”, and “fatty acids”. (**Figure 9B**). In light of this, we named cluster A as “metabolic phenotype, C as “immune phenotype”, and B as “mixed phenotype”. These results indicated that fibrotic livers presented crosswise variances and similarities; accordingly, classification into three clusters might help distinguish different phenotypes.

### 3.7 Upstream regulators of core DEGs and their expression evolution along liver pathology

**Figure 10:**
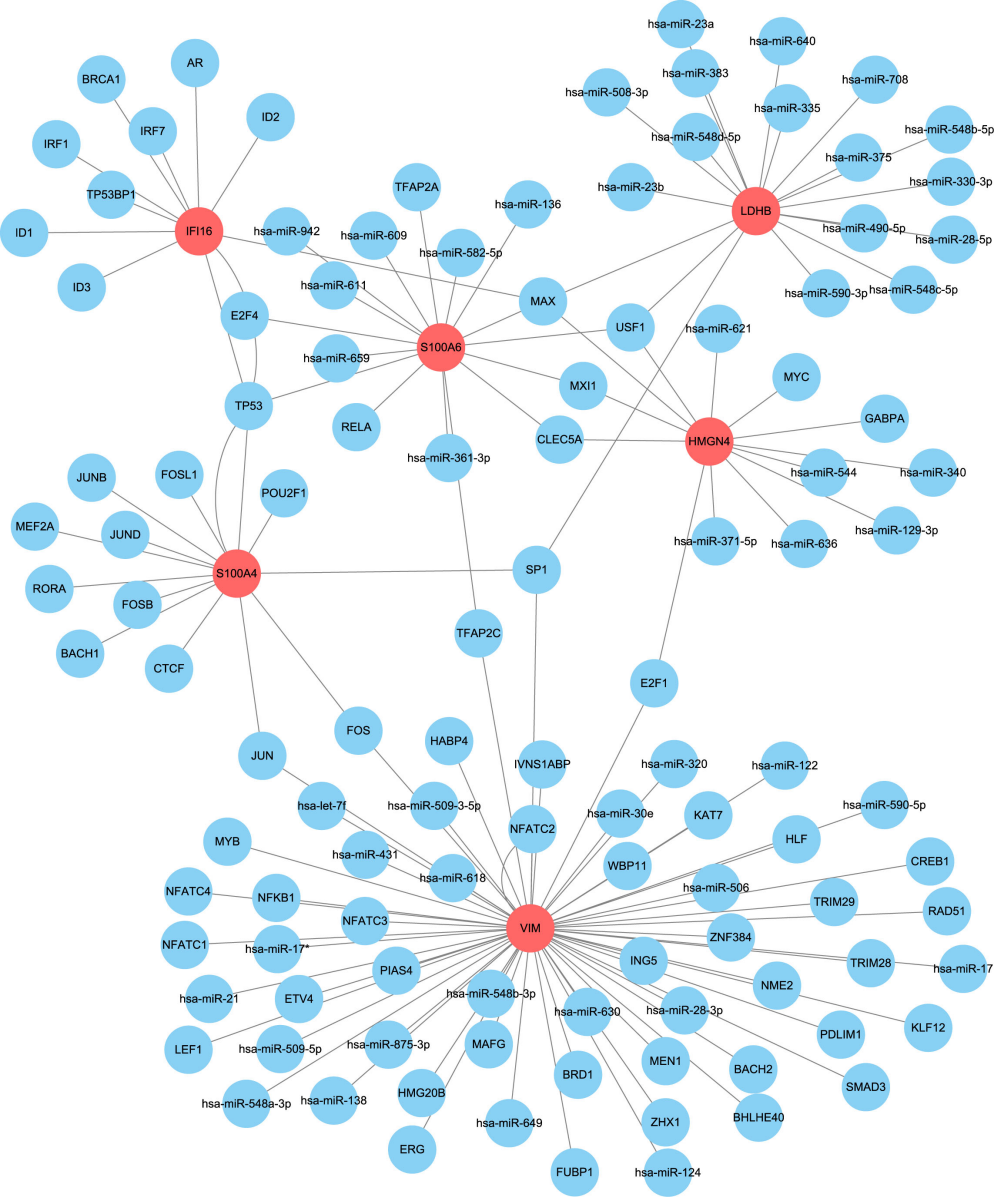
Upstream regulatory network of core DEGs. Core DEGs (red) and their predicted upstream regulators (blue) in the regulatory network constructed by Cytoscape software.

**Figure 11:**
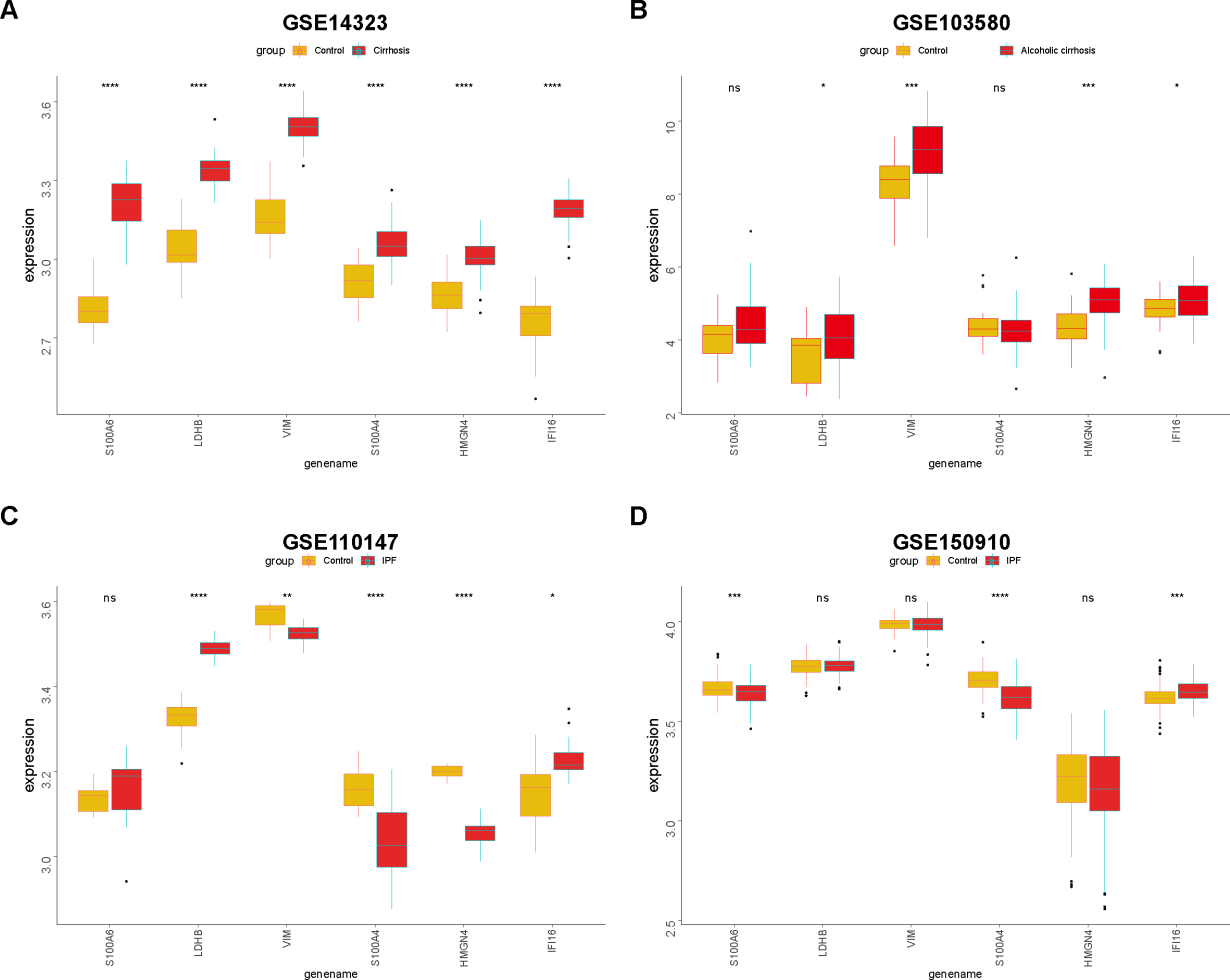
Expression of core DEGs in fibrosis, cirrhosis, and IPF. Differences in core DEG expressions in HCV-related cirrhosis **(A)**, alcoholic cirrhosis **(B)**, and IPF **(C, D)** were compared with the control. *p<0.05, **p<0.01, ***p<0.005, ****p<0.001, and ns, non-significant.

With the help of the regnetwork database(https://regnetworkweb.org/) and cytoscape software, we mapped the regulatory network of core DEGs in liver fibrosis (**Figure 10**). Core DEGs shared several transcriptional factors, namely E2F4, MAX, TP53, USF1, MXI1, CLEC5A, SP1, E2F1, and JUM. As liver fibrosis arises from various etiologies and leads to cirrhosis once it progresses towards irreversible stages, we examine the roles of core DEGs in different etiologies. By comparing their expression in HCV- and alcohol-related cirrhosis, we found that most of the core DEGs were significantly upregulated in HCV- and alcohol-related cirrhosis; specifically, S100A4 and S100A6 were upregulated in HCV-related cirrhosis but presented comparable expression in alcoholic cirrhosis and control livers (**Figures 11A, B**). These results indicated that they might exert distinct functions in liver fibrosis of different origins. As for fibrosis in other tissue types, idiopathic pulmonary fibrosis (IPF), for instance, manifested another expression pattern of the core DEGs against liver cirrhosis. S100A6, VIM, S100A4, and HMGN4 were downregulated or unchanged, while LDHB was upregulated or remained unchanged in IPF. Only IFI16 was upregulated in both IPF and liver cirrhosis, indicating that it was universally activated in fibrosis (**Figures 11C, D**).

### 3.8 Tumorigenic roles of core DEGs

**Figure 12:**
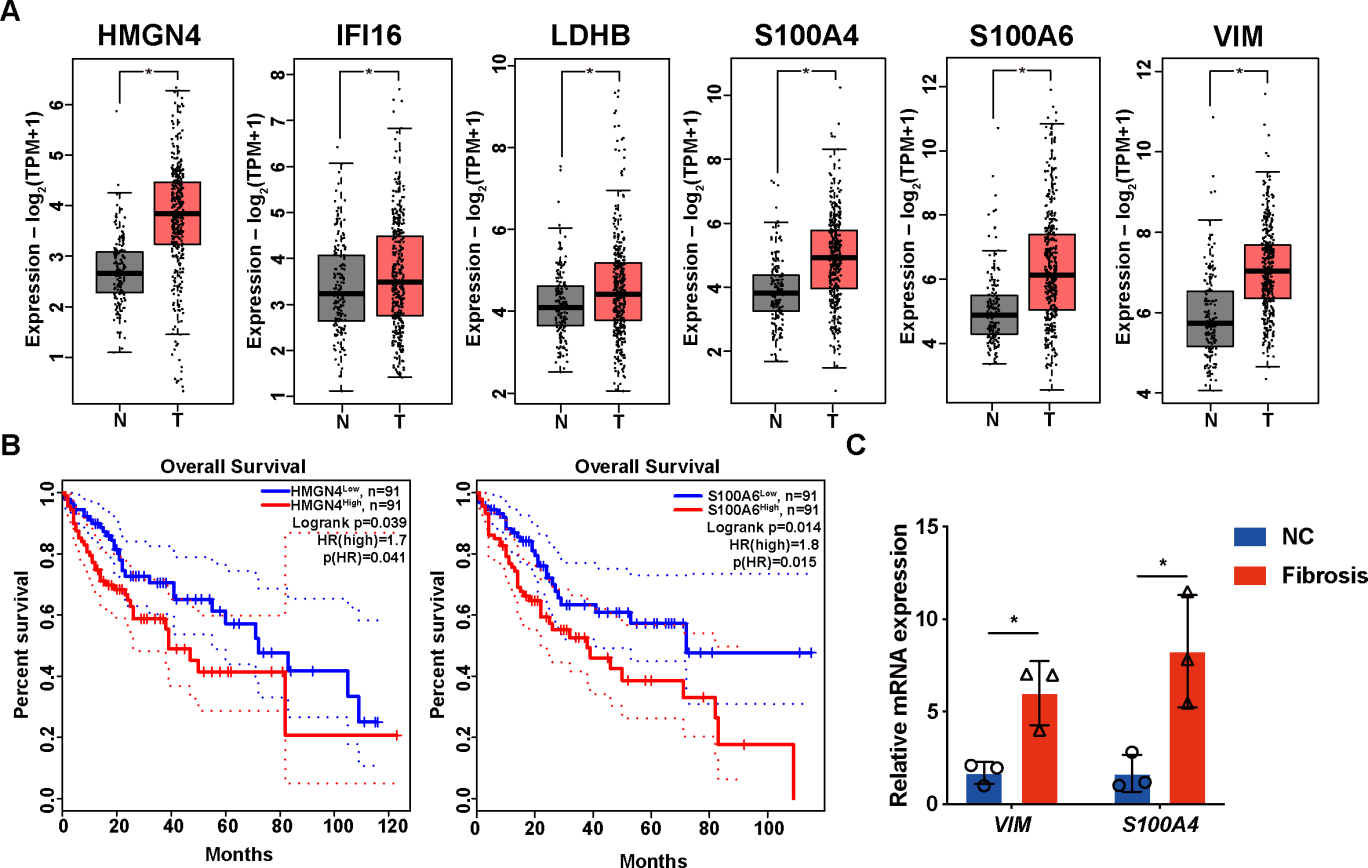
Expression and prognostic values of core DEGs in patients with HCC. **(A)** expressions of core DEG in the TCGA-LIHC patients. **(B)** overall survival of HCC patients with high or low expression of core DEGs. Only genes with significant association with survival are displayed. **(C)** mRNA expressions of VIM and S100A4 in normal and fibrotic mice detected by RT-qPCR. *p<0.05.

As the core DEGs function universally in fibrotic pathology, we hypothesized that they might participate in fibrotic progression to liver tumors as well. By comparing their expression in tumor and non-tumor tissues from the TCGA-LIHC database, we found that all core DEGs were upregulated in tumoral specimens (**Figure 12A**); moreover, HMGN4 (log-rank *p*= 0.039) and S100A6 (log-rank p= 0.014) expressions in HCC patients were significantly correlated with their overall survival (**Figure 12B**), suggesting their tumorigenic potential in liver fibrosis progression.

VIM and S100A4 were upregulated in liver fibrosis but downregulated in IPF; we further collected mice fibrotic livers to examine their expression. As expected, fibrotic mice presented higher expression of VIM and S100A4 in the liver than normal mice (**Figure 12C**).

## 4 Discussion

Staging of liver fibrosis currently relies upon liver biopsy and noninvasive imaging is increasingly applied for screening and diagnosis (29, 30). Fibrosis staging indicates the severity of the condition and the study proposed a model for fibrosis classification that also reflected the immune and metabolic status of the nidus. We screened lactylation-related core DEGs between mild and advanced fibrosis and discovered that fibrosis stages are associated with immune infiltration. One of the core DEGs, IFI16, might also play certain roles in lung fibrosis as suggested by its upregulation in IPF.

The model integrated the feature of disease progression with immunity and metabolism as constructed with concerns of fibrotic stage and lactylation activity. The phenotypes cataloged by the model exhibit varied inclinations in immune infiltration and metabolic reprogramming. The metabolic phenotype features activated lipid metabolism, the immune phenotype involves more immune components, and the mixed phenotype stands as the intermediated state of both clusters. As early-stage samples made up the majority of the metabolic phenotype and advanced fibrosis mainly fell under the immune phenotype, we inferred that the main cause of fibrotic pathology begins at lipid metabolic reprogramming and turns to immune response, which might enlighten our presupposition of fibrosis progression and direct further investigation.

The study found that immune infiltration exhibited distinct patterns in mild and advanced fibrosis (**Figure 5B**). Besides the relay of metabolic reprogramming and immunity, the discrepancy might also result from the deterioration of etiology such as the progression of MAFLD to MASH (4, 31, 32). B cells, CD4 T cells, CD8 T cells, and dendritic cells were activated in advanced fibrosis, accompanied by increased infiltration of γδ T cells, MDSCs, and natural killer T cells (NKTs), and most of these are established pro-fibrosis immunocytes (31, 33, 34). In contrast, macrophages and neutrophils, mediators of tissue repair (31), were reduced in proportion during disease advancement. These findings not only correspond to previous reports but also summarize cell types with pro/anti-fibrotic functions.

In corroboration of our hypothesis that lactylation might influence the infiltration and activation of immune cells, core DEGs sifted from lactylation-related genes demonstrated significant correlations with immune infiltration and functioning (**Figures 5C**, **7**). Lactylation-related core genes were subjected to ssGSEA (**Figure 5C**) and GSEA (**Figure 7**) to examine the pathways mediating the crosstalk between gene expression and immune cell infiltration. For example, almost all core DEGs were found to be closely related to activated CD4 T cells, natural killer T cells, MDSCs, regulatory T cells, and so forth (**Figure 5C**). In the GSEA results (**Figure 7**), “Immunoregulatory interactions between a Lymphoid and a non-Lymphoid cell” and “Interferon Signaling” pathways, which involve the interaction between the above cells and mediate the killing activity of T cells, were enriched for the core DEGs. Therefore, lactylation might modulate the inclination between immune killing and immune regulation through these core DEGs. Histone lactylation has been widely documented with immunoregulatory functions. For instance, glycolysis was boosted by STAT5 (signal transducer and activator of transcription 5A) in acute myeloid leukemia (AML) cells to provide excessive lactide for lactylation of PD-1, making tumor cells susceptible to immune checkpoint inhibitors (ICIs) (35). Activated glycolysis facilitated H3K18la lactylation at the promoter region of FOXP3 to compromise the immune killing of NKT-like cells (36). Our analysis of immune infiltration in liver fibrosis unveiled the association of lactylation with mast cells and MDSCs, which has barely been reported. There is a high probability that lactylation extensively functions in all kinds of immunocytes to participate in their immune responses, which are commonly accompanied by activated glycolysis (9, 37). Thus, research to expand our knowledge on the crosstalk between lactylation and immunocytes and delineate the underlying mechanisms might be promising in the field of immunoregulation.

In addition, lactylation might have universal impacts on fibrotic pathologies. Most core DEGs were upregulated in HCV-related and alcohol-related cirrhosis except S100A4 and S100A6 (**Figures 11A, B**). Though sharing some common pathology concerning inflammation (38), fibrosis originating from hepatitis viral infection or alcohol abuse might manifest some inclination to immune response, lipid toxicity, and oxidative stress (39). Thus, a variety of gene functions in cirrhosis with different etiologies is not uncommon. For example, the genetic variant rs738409 (G) in the PNPLA3 gene was an independent risk factor for the development of HCC in patients with alcoholic cirrhosis but showed no influence in the progression of HCV-related cirrhosis to HCC (40). At the same time, fibrosis in the liver and lungs also share certain functional molecules such as TGF-β (41) and serotonin (5-hydroxytryptamine(5-HT)) (42). In the study, we found that most core DEGs manifested dysregulation in IPF, especially IFI16, the expression of which was increased in both validation datasets (**Figures 11C, D**). IFI16 has been previously reported to function in lung cystic fibrosis (43), while its role in liver fibrosis has scarcely been researched (44). Therefore, our data excavated a profibrotic gene with pan-tissue potential.

Last but not least, the expression of the core DEGs also reflected the tumorigenic risks underlying liver fibrosis (**Figure 12**). HCC is one of the most unfavorable outcomes of liver fibrosis and cirrhosis, and there might be shared molecules that function throughout fibrosis to tumorigenesis. For example, depletion of Apobec1 complementation factor (A1CF) in a mouse model upregulated genes responsible for oxidative stress, inflammatory response, extracellular matrix organization, and proliferation, resulting in spontaneous fibrosis, dysplasia, and HCC (45). On one hand, genes with accepted fibrotic roles might exert certain functions in hepatocellular carcinoma; on the other hand, our results underscore the consistent involvement of lactylation in liver fibrosis advancement and HCC development (46, 47). During the process, immunocytes might be the main executive component (48). Therefore, it would be worth investigating the roles of lactylation-associated immune infiltration in liver fibrosis progression to HCC for precise treatment (49, 50).

In summary, the study constructed a phenotyping model of liver fibrosis with lactylation-related DEGs between early- and later-stage patients, which can classify the cases into metabolic, immune, and intermediate clusters as well as predict the tumorigenic potential of liver fibrosis. The distinct inclination of the clusters revealed the interplay of metabolism and immunity in the progression of fibrotic pathology. To what extent these two forces function at different stages of liver fibrosis and how they are poised for HCC development may be interesting propositions in future investigations.

## Data Availability

The original contributions presented in the study are included in the article/supplementary material. Further inquiries can be directed to the corresponding authors.
